# eHealth in Modern Patient-Caregiver Communication: High Rate of Acceptance Among Physicians for Additional Support of Breast Cancer Patients During Long-Term Therapy

**DOI:** 10.2196/cancer.5132

**Published:** 2016-09-19

**Authors:** Thomas Kirkovits, Timo Schinkoethe, Caroline Drewes, Caroline Gehring, Ingo Bauerfeind, Nadia Harbeck, Rachel Wuerstlein

**Affiliations:** ^1^ Breast Center, University Hospital Munich Department of Gynecology and Obstetrics Comprehensive Cancer Center Ludwig Maximilian University Munich Germany; ^2^ GEMKOM - Gesellschaft für moderne Kommunikation in der Medizin Munich Germany; ^3^ Breast Center, Landshut Hospital Department of Gynecology Landshut Germany

**Keywords:** eHealth, mobile health, telemedicine, physicians, acceptance, breast cancer, patient compliance, patient adherence

## Abstract

**Background:**

Lack of adherence and compliance with drug regimens among breast cancer patients represent substantial problems in oral therapies, leading to significant impacts on mortality. Where other systems have failed, electronic health (eHealth) could be a possible solution to improve medication intake, along with the doctor-patient relationship. Initial results from studies concerning new interventions for therapy support are promising, but reports suggest that general acceptance of new treatment support tools is needed among patients and physicians alike.

**Objective:**

The aim of this study was to investigate the actual use of the Internet and other modern media among physicians involved in breast cancer treatment.

**Methods:**

Using a standardized questionnaire, actual utilization of new media among physicians was analyzed. Internet-related behaviors in private, as well as in business life, were investigated. Attention was focused on physicians’ opinions regarding modern eHealth tools and how patients could be best supported to enhance adherence.

**Results:**

A total of 120 physicians, all participating in breast cancer care, completed the questionnaire (median age 41 years). Almost all participants (99.2%, 119/120) used the Internet for general purposes and 98.3% (118/120) used it for medical issues as well. Virtually all medical professionals (99.2%, 119/120) reported that they owned a computer, while more recently invented technologies such as tablets and smartphones were owned by 31.9% (38/119) and 73.1% (87/119), respectively. The Internet was favored by 66.4% (79/119) of the physicians in our survey as a source for patient support; 71.2% (84/118) would also favor modern media for side effect registration. Based on our analysis, the most frequent Internet-utilizing physicians were characterized by age <60, worked in a hospital, and were employed as a junior physician.

**Conclusions:**

This study demonstrated a high usage of Internet-related technologies among physicians, indicating that the use of eHealth for advanced and individualized support in breast cancer care is a promising addition to treatment management. Such technologies have the potential to enhance adherence and compliance in therapy among cancer patients.

## Introduction

The Internet has become increasingly relevant for daily use across the globe. General Internet usage among the population of the European Union has increased from 49% in 2006 to 72% in 2013 [1]. In total, approximately 3 billion people around the world used the Internet in 2014, which constitutes an increase of 0.6 billion users since 2012 [2,3].

As reported in 2003, 4.5% of all queries performed on the Internet concerned health-related issues, which represented approximately 67.5 million health-related searches being performed every day [4]. Electronic health (eHealth) and mobile health, as modern mechanisms of patient management, are enhancing the support systems for many different diseases [5,6].

eHealth applications are being successfully used in industrialized countries. Particularly in chronic diseases, such as hypertension, heart failure, or chronic obstructive pulmonary disease, many studies demonstrate that patients could benefit from using online eHealth support systems [7-10]. Many eHealth-related research studies have indicated that patients suffering from diabetes mellitus demonstrate positive, and often significant, improvements in therapy [11-13]. As reported by Appel et al, in the case of obesity among adults, weight loss in the interventional group (which was assigned to different types of online support or assistance by telephone) was higher than in the control group without such support. Reducing the body mass index among participants for 2 years demonstrated that eHealth systems also work over longer time periods [13]. In countries with few resources concerning medical care (eg, Nigeria), mobile phones are being used to improve the health status among cancer patients who cannot afford to visit their doctor every day [14].

A recently published article concerning breast and prostate cancer reported that patients experienced significantly lower symptom distress by using eHealth modalities compared to the control group [15]. Kuijpers et al reported that the usage of eHealth may not just work for chronic diseases, but may also have a positive effect on empowerment and physical activity (and therefore on quality of life) for cancer patients [16].

Breast cancer had a worldwide incidence of approximately 1.67 million in 2012, and is one of the most likely causes of death among women [17]. The implementation of antihormonal medication in endocrine-sensitive disease, or targeted therapies for patients with a human epidermal growth factor receptor 2-positive carcinomas, brought significant new treatment options for women suffering from breast cancer [18]. These advances have resulted in patients undergoing oral treatments for ten years or more, during which time patients have minimal contact with their physicians.

Hershman et al were the first group to specify that early discontinuation and nonadherence during oral antihormonal therapy with tamoxifen or aromatase inhibitors had a significant impact on mortality. Among all patients who were included in the study (8769), 31% discontinued therapy (early discontinuation, meaning patients discontinued therapy after 180 days elapsed from prior prescriptions) [19]. The Patient's Anastrozole Compliance to Therapy program also reported that even adding special educational materials to standard patient information did not have a significant positive effect on compliance or persistence with adjuvant endocrine therapy in breast cancer care [20]. However, some studies using eHealth interventions to enhance adherence have demonstrated promising results regarding improvements for these problems [21]. These trials suggest that using an eHealth-based support system may have a positive effect on compliance and adherence among breast cancer patients.

Before attempting to improve the connection between physicians and their patients by using the Internet during medical care, and to ameliorate the treatment of early and metastasized breast cancer, it is necessary to first investigate physicians’ general attitudes concerning new media and Internet usage. Gund et al indicated that the majority of the health care professionals queried in their study had a positive attitude towards current and future eHealth tools for out-of-hospital care for patients with chronic heart failure [22]. The Telemedical Interventional Monitoring in Heart Failure trial also indicated that eHealth acceptance among medical professionals was very high [23]. The authors also reported that it is necessary for implementation of online support systems to evaluate the acceptance among patients, but also among physicians who have a central role in disease treatment [23].

The aim of this survey was to investigate Internet usage behaviors, and usage of other modern media (eg, smartphones), among health care professionals who were involved in the treatment of patients suffering from breast cancer. This study examined how physicians were equipped with electronic devices (eg, computers, mobile phones) and how they used them in general, and for medical matters in particular. This study also examined physicians’ opinions regarding future eHealth applications, and the personal demographic information given by the participants (eg, age, qualification).

## Methods

This study was submitted and accepted by the local ethics committee of the medical faculty of Munich University.

### Participants

Physicians involved in breast cancer treatment were invited to participate in our survey. The questionnaire was handed out to participants on two occasions in 2012: First at the COMBATing Breast Cancer conference in Munich, Germany, and later at a breast cancer-specific meeting organized by Tumor Center Munich. A paper-based questionnaire was provided for each participant. No individuals were excluded from the survey; all participants were given the opportunity to fill in the questionnaires voluntarily and anonymously.

### Questionnaire

The German-language questionnaire contained 4 sheets; three listed questions and one contained information about the survey. The questionnaire was designed by the study investigators (Ludwig-Maximilians-Universitat Munich Breast Center). No physician belonging to the study group completed the questionnaire, in an effort to prevent bias.

The questionnaire contained 33 items in total, separated into 3 sections. In the first section, participants were asked for general demographic information, including age, sex, place of residence and employment, year of examination, current qualifications, and medical specialty. The second section examined Internet usage in general and focused on participants’ habits using the Internet for health-related topics. Participants were also interviewed regarding their possession of computers, mobile phones, and other electronic devices, how they use these technologies, and who is allowed to use these media at their place of employment (physicians only, or nurses and other co-workers). In the third section, the medical professionals were asked to state their opinions on future eHealth tools: part one asked about a telephone-hotline, which cancer patients could turn to for support; the second part contained questions focused on future support for patients using the Internet or smartphones, and collecting information regarding side effects of therapy via electronic devices. The responses for this part could be rated on a five-point scale from *agreement* (1) to *denial* (5).

Anonymity was assured by not collecting personal information such as names or birth dates. In each questionnaire, participants were asked to complete every single question, even though this was not absolutely necessary for data analyses. Each participant completed the survey once during one of the meetings. During each convention, participants were given written and verbal instructions to refrain from completing the questionnaire twice.

### Statistical Analyses

Results were analyzed using IBM SPSS-Statistics. Student t-tests were used to examine differences between groups. To describe disparities between different parameters, odds ratios were used and *P*-values were computed with a level of significance <5%.

The questions in the questionnaire could be answered as single- or multiple-choice answers. In the third section, which focused on opinions regarding future eHealth tools, there were single answer possibilities of Grades 1 (*agreement*) to 5 (*denial*). For analytical purposes, Grades 1 and 2 were collected together as *agreement*, Grade 3 as *neutral*, and Grades 4 and 5 as *denial*.

Subgroups were formed to further examine which type of physician was most likely and willing to use the Internet or smartphone-related health support systems. Significant differences were evaluated, taking into account gender, age groups, medical facilities, qualifications, and size of the city in which physicians were employed.

## Results

### Participants’ Sociodemographic Characteristics

A total of 120 active medical professionals completed the questionnaire, out of 154 participants who attended one of the events in which data was collected. The median age of participants was 41 years, and the cohort was 57.5% (69/120) female and 40.8% (49/120) male (Table 1). More than half (60.8%, 73/120) of all participants worked in a hospital institution; of these, 65.7% (48/73) worked in a university hospital. Approximately one quarter (26.7%, 32/120) of respondents were employed in any sort of practice. Approximately two thirds of the attendees were gynecologists (68.3%, 82/120), and 46.7% (56/120) were treating patients as gynecological oncologists. A small number of physicians with origins in other medical specializations completed the questionnaire (hematologists and oncologists: 9.2%, 11/120; radiation therapists: 7.5%, 9/120; radiologists: 1.7%, 2/120). All participants were German, which was assured by reporting the region of Germany in which participants were employed.

**Table 1:** Physicians’ sociodemographic characteristics.

Participants’ characteristics		%	n/N
**Gender**			
	Female	57.5	69/120
	Male	40.8	49/120
	Not available	1.7	2/120
Median age in years (range)		41 (25-68)	
**Age**			
	20-29	16.8	19/113
	30-39	23.9	27/113
	40-49	30.1	34/113
	50-59	22.1	25/113
	60-69	7.1	8/113
**Current qualification**			
	Medical student	2.5	3/120
	Junior physician	16.7	20/120
	Board certified specialist	26.7	32/120
	Senior physician/head of department	21.7	26/120
	Chief physician	17.5	21/120
	Others	6.7	8/120
	Not available	8.3	10/120
**Interdisciplinary specialization**			
	Oncology (gynecological specialist)	46.7	56/120
	General gynecology	21.7	26/120
	Hematology and oncology	9.2	11/120
	Radiation therapy	7.5	9/120
	Radiology	1.7	2/120
	General surgery	0.0	0/120
	Others	9.2	11/120
	Not available	4.2	5/120
**Medical facility**			
	Ambulatory/private practice	25.8	31/120
	Medical care center	0.8	1/120
	General hospital	5.0	6/120
	Hospital with high grade of specialization	1.7	2/120
	Teaching hospital	14.2	17/120
	University hospital	40.0	48/120
	Others	5.0	6/120
	Not available	7.5	9/120

### Usage of Modern Media and Internet

Almost all participants used a telephone and 73.1% (87/119) of the participants owned a smartphone (Table 2). The ownership of private computers among physicians was very high. The most favored computer was the notebook (83.2%, 99/119), followed by 60.5% (72/119) of physicians who were in possession of a desktop personal computer. Tablet computers were owned by 31.9% (38/119) of the participants. Above age 60, the percentage of physicians owning a tablet declined to less than 12.5% (1/8). With regards to more recently developed devices, smartphones were owned by 71.3% (75/105) of the participants younger than 60, and by 50% (4/8) of those above 60 years of age. Most physicians (89.7%, 105/117) used the Internet at work, and in most cases nurses and physicians’ assistants were able to use the Internet at work (74.6%, 88/118).

**Table 2:** Actual usage of modern media by breast cancer specialists.

Technology and media		%	n/N
**Phone**			
	Any type of phone	99.2	119/120
	Landline phone	89.9	107/119
	Any mobile phone	100.0	119/119
	Mobile phone (no Internet)	47.1	56/119
	Apple iPhone	53.8	64/119
	Smartphone using Android	13.4	16/119
	Smartphone (others)	5.9	7/119
**Private computer**			
	Any type of computer	99.2	119/120
	Personal computer with Internet access	60.5	72/119
	Notebook with Internet access	83.2	99/119
	Apple iPAD	26.9	32/119
	Tablet using Android	5.0	6/119
	Tablet (others)	0.0	0/119
**Internet access at workplace**			
	Internet access	89.7	105/117
	Divided patient-network and Internet access	10.3	12/117
	No Internet access	0.0	0/117
**Internet usage at workplace**			
	Physicians	97.5	115/118
	Nurses/physicians’ assistants	74.6	88/118
	Other coworkers	66.1	78/118
**Mobile phone usage**			
	Always with me	78.0	92/118
	Only en route	16.1	19/118
	Mostly at home	0.8	1/118
	Mostly out of use	5.1	6/118
**Smartphone usage**			
	Business and private	90.9	80/88
	Private only	9.1	8/88
	Business only	0.0	0/88

Almost all (99.2%, 119/120) participants used the Internet for general purposes (Table 3). Approximately 84.9% (101/119) of medical professionals used the Internet in their daily routine, while the remaining 15.1% (18/119) used the Internet more than once per week. The majority of respondents took advantage of the Internet at home as well as at work. For health-related issues, the Internet was used by 98.3% (118/120) of physicians, and a smartphone was used by 38.1% (45/118) of respondents. Email communication was the most frequently used function concerning the Internet, followed by reading online news or articles. Approximately one third of the participants (35.8%; 43/120) used the Internet for social networks and 31.7% (38/120) used the Internet for making calls via computer. Concerning health-related platforms, PubMed was the most used resource among physicians, followed by online guideline search (84.2%, 101/120) and Google (79.2%, 95/120).

**Table 3:** Internet usage among breast cancer specialists.

Usage		%	n/N
Internet usage in general		99.2	119/120
**Frequency of Internet use**			
	Daily	84.9	101/119
	>Once per week	15.1	18/119
	<Once per week	0	0/119
**Site of Internet use**			
	At home only	0.9	1/115
	At work only	2.6	3/115
	Both	96.5	111/115
**Types of Internet use**			
	Email	98.3	118/120
	Reading news/articles online	66.7	80/120
	Wikis/online encyclopedia	60.0	72/120
	Gain health information	41.7	50/120
	Social networks (private)	35.8	43/120
	Making calls via the Internet	31.7	38/120
	Educational online courses	22.5	27/120
	Social networks (business)	17.5	21/120
**Types of Internet use (health-related platforms)**			
	PubMed	87.5	105/120
	Online guideline search	84.2	101/120
	Google	79.2	95/120
	Adjuvant!	57.5	69/120
	Wikipedia	52.5	63/120
	Rote Liste (collection of all medications available)	48.3	58/120
**Internet usage for health-related issues**			
	Yes	98.3	118/120
	No	1.7	2/120
**Smartphone usage for health-related issues**			
	Yes	38.1	45/118
	No	61.9	73/118

### Future Use of eHealth Tools

The desire to support patients via new media was accepted by the majority of participants. Table 4 gives a view of physicians’ opinions regarding future eHealth tools. Grades 1 and 2 were summed, and therefore demonstrate *agreement*, Grade 3 demonstrates *neutral*, whereas Grades 4 and 5 represent *denial*.

Two-thirds of respondents (66.4%, 79/119) favored the option of their patients using the Internet as a source of support, while more than half (51.3%, 60/117) favored therapy assistance via smartphone. The online registration of side effects via new media was favored among the majority of physicians (71.2%, 84/118).

If a system existed for the online registration of side effects, most respondents would want to be informed about problems concerning their patients’ treatment via email (43.2% *agreement*, 51/118; vs 28% *denial*, 33/118) or via Internet-based platforms (35.7% *agreement*, 40/112; vs 33.0% *denial*, 37/112). Phones and fax machines were disliked for receiving information about side effects (phones: 14.0% *agreement*, 16/114; vs 67.5% *denial,* 77/114. Fax machines: 7.9% *agreement*, 9/114; vs 69.3% *denial*, 79/114).

**Table 4:** Physicians’ opinions regarding future use of eHealth tools.

Description	Total (N)	Agreement, % (n)		Neutral, % (n)	Denial, % (n)	
		Grade 1	Grade 2	Grade 3	Grade 4	Grade 5
Support for patients via Internet	119	28.6 (34)	37.8 (45)	22.7 (27)	0.8 (1)	10.1 (12)
Support for patients via smartphone	117	24.8 (29)	26.5 (31)	25.6 (30)	9.4 (11)	13.7 (16)
Registration of side effects via electronic devices	118	31.4 (37)	39.8 (47)	12.7 (15)	7.6 (9)	8.5 (10)
Getting information about side effects via email	118	17.8 (21)	25.4 (30)	28.8 (34)	7.5 (9)	20.0 (24)
Getting information about side effects via Internet	112	9.8 (11)	25.9 (29)	31.3 (35)	8.0 (9)	25.0 (28)
Getting information about side effects via phone	114	6.1 (7)	7.9 (9)	18.4 (21)	19.3 (22)	48.2 (55)
Getting information about side effects via fax	114	2.6 (3)	5.3 (6)	22.8 (26)	14.9 (17)	54.4 (62)

To further examine how physicians wanted to receive information regarding side effects (Table 5), only the participants who *accepted* or *disapproved* new forms of communication were considered. The majority of medical professionals who accepted online side effect registration preferred to be informed via email or the Internet.

**Table 5:** Acceptance of documentation of side effects via new media.

Delivery method of registered side effects	Acceptance of side effect documentation, % (n/N)	Disapproval of side effect documentation, % (n/N)
Via email	53.0 (44/83)	36.8 (7/19)
Via Internet	46.8 (36/77)	21.1 (4/19)
Via phone	12.7 (10/79)	15.8 (3/19)
Via fax	10.0 (8/80)	26.3 (5/19)

### Correlations and Further Analyses

To determine if there was a typical type of physician whose affinity for new media was particularly high or low, further evaluations were undertaken (Table 6). High rates of acceptance for Internet support were evident among physicians up to the age of 60. Above age 60 there was a distinct drop in acceptance levels, although acceptance rates never declined lower than 50% in the older age group. Concerning the acceptance of online side effect registration among physicians, acceptance decreased with increasing age, with the lowest approval rates in the 50-59 age group (68.4%, 13/19). With regards to medical professionals favoring smartphones for patient support, the highest percentage (90.5%, 19/21) was found in the 30-39 age group.

According to the physicians’ place of employment, those who worked in hospitals (general or university hospital) preferred support and side effect documentation via the Internet more than physicians working in out-patient practices. Concerning participants’ grade of qualification, junior physicians were the most likely to use new media for patient support regarding all three eHealth methods (Internet, smartphones, and online side effect registration). Gender was not a factor that influenced physicians’ opinions on Internet support. Further analysis indicated that physicians who owned a smartphone were more willing to support their patients online (odds ratio 1.70, 95% CI 1.32-20.25, *P*=.012) than physicians who were not in possession of such technology.

**Table 6:** Subgroup-specific results for supporting patients via eHealth tools.

Characteristics		Acceptance of support via Internet, % (n/N)	Acceptance of support via smartphone, % (n/N)	Acceptance of side effect registration, % (n/N)
**Sex**				
	Female	85.5 (47/55)	65.5 (36/55)	80.0 (48/60)
	Male	86.5 (32/37)	77.1 (27/35)	84.1 (37/44)
**Age**				
	20-29	88.2 (15/17)	73.3 (11/15)	88.2 (15/17)
	30-39	95.7 (22/23)	90.5 (19/21)	88.0 (22/25)
	40-49	85.7 (24/28)	66.7 (18/27)	81.3 (26/32)
	50-59	81.3 (13/16)	65.0 (13/20)	68.4 (13/19)
	60-69	66.7 (4/6)	33.3 (1/3)	71.4 (5/7)
**Medical facility**				
	Practice	78.3 (18/23)	68.2 (15/22)	76.0 (19/25)
	Hospital	89.5 (17/19)	63.2 (12/19)	86.4 (19/22)
	University hospital	87.2 (34/39)	73.7 (28/38)	82.2 (37/45)
**Workplace city size**				
	Less than 1000	0.0 (0/0)	0.0 (0/0)	0.0 (0/0)
	1000-9999	100.0 (2/2)	100.0 (2/2)	100.0 (3/3)
	10,000-49,999	75.0 (12/16)	40.0 (6/15)	64.3 (9/14)
	50,000-99,999	83.3 (5/6)	83.3 (5/6)	100.0 (7/7)
	More than 100,000	88.4 (61/69)	75.8 (50/66)	82.5 (66/80)
**Qualification**				
	Medical student	100.0 (3/3)	66.7 (2/3)	66.7 (2/3)
	Resident physician	100.0 (18/18)	88.2 (15/17)	94.7 (18/19)
	Medical specialist	90.9 (20/22)	72.7 (16/22)	82.1 (23/28)
	Senior physician	80.0 (16/20)	59.1 (13/22)	82.6 (19/23)
	Chief physician	86.7 (13/15)	71.4 (10/14)	76.5 (13/17)
	Others	42.9 (3/7)	40.0 (2/5)	60.0 (3/5)

## Discussion

The aim of this study was to describe Internet usage behaviors among breast cancer physicians, and to evaluate their opinions regarding future eHealth applications that may further improve breast cancer treatment. Many surveys have already demonstrated that it is possible to implement a patient support system using eHealth [7,10-13,24-31], but little is known about physicians’ acceptance of such technologies. Our study provides information regarding actual Internet usage and modern media habits among gynecological oncologists, breast cancer specialists, and other physicians treating patients with breast cancer. Many surveys are being conducted in specialized centers that already make use of eHealth technologies, and therefore these physicians are considered to be interested in using modern media to communicate with their patients.

This study focused on breast cancer and oncological specialists employed in different work settings (practices and clinics), and participants were not considered to have had a great deal of experience using eHealth technologies. Future improvements in the management of early and metastatic breast cancer may benefit from physicians’ acceptance of new media, in addition to the patients using these resources themselves.

When examining general Internet usage among the participants (Table 3), almost all respondents were Internet users (99.2%, 119/120). This finding is comparable to another international study that examined physicians’ characteristics regarding online database usage in regional hospitals (99.6%) [32]. Compared to the general population of Germany (where this survey was conducted), the incidence of daily Internet use (84.9%, 101/119) was higher among participants than the general population (81%) [33]. Email was favored as a communication tool by 98.3% (118/120) of survey respondents (compared to 93% of the general German population), while a similar percentage (66.7%, 80/120; vs 68%) used the Internet for reading news or articles online [33].

Examination of participants’ and the general population’s possession of new media indicated that a similar percentage of people owned any type of phone (99.2%, 119/120 vs 99.7%) [34]. The rate of computer ownership among medical professionals was 99.2% (119/120), which was higher than the general German population (85%) [34]. General Internet usage was lower among Germany’s population (daily use 76%) compared to the participants in this survey (84.9%, 101/119) [35]. In contrast, the participants in our study belonged to a *high end collective* that uses the Internet and different media in their daily practice, as well as for conducting trials. It is assumed that this population owns more electronic devices (and may have more experience in using the Internet) than the general German population. By attending breast cancer-specific meetings (where this study was conducted), participants were considered to be very interested in general research, and therefore might have interests in modern media for patient support. This study only reflects the indicated use of modern media in a specific cohort of participants, which limits the broader applicability of our findings.

Although the questionnaires for this study were completed in 2012, we consider our findings to be up-to-date. General Internet usage (daily use or more than once per week) among German employees increased only 1% from 2012 to 2014 (from 96% in 2012 to 97% in 2014) [36]. Regarding the use of a computer, the percentage of the German population that owned a computer increased from 79% in 2012 to 82% in 2014 [37]. These facts indicate that there should not be a substantial change in percentages now, although we cannot easily calculate these data.

In addition to the high percentage of physicians using the Internet and being interested in future eHealth support, data was provided that 25.4% (30/118) of nurses or physicians’ assistants had no access to the Internet in their workplace. Regarding this issue, we have conducted another survey, which will display nurses' opinions on eHealth and modern media use.

The typical physician that is most likely to use modern media for patient support and online therapy assistance (according to the data compiled in this study) is characterized by age <60, working in a hospital, and having the position of a junior physician (Table 6). Other characteristics (eg, gender or the population of the physicians’ city) did not have an impact on opinions concerning future eHealth solutions. Chiu et al reported that age <50 is a significant factor for the usage of online databases, and that having a faculty position is a significant factor concerning online database usage [32]. This effect was not present in our study. Chiu et al also demonstrated that gender does not affect the likelihood of using the Internet for gaining information [32].

Our study indicates that there is a great deal of interest among physicians for implementing online support systems to provide additional therapy assistance. Oncologists who already owned smartphones were more willing to support their patients using this type of media for therapy management than medical professionals who did not own a smartphone. This finding indicates that physicians who are already in possession of modern media are more likely to utilize eHealth.

Implementing new eHealth tools could lead to increased adherence and compliance, reduced health care costs, and consequently to improvements in breast cancer survival, as taking medication regularly is an important factor concerning mortality [19]. eHealth may also help oncologists monitor potential side effects more directly, and thus give physicians the chance to react immediately. Such advances have the potential to improve the doctor-patient relationship and communication between breast cancer patients and their health care teams.

Further studies investigating the opinions of other occupational groups working in breast cancer treatment (eg, nurses, psychologists) regarding future inventions will be useful to introduce a more personalized, patient-oriented approach for managing side effects. Furthermore, clinical trials using eHealth support in breast cancer therapy management are needed to investigate the actual usage of modern media and supportive tools, and their impacts on compliance and outcomes. Therefore, our working group is currently developing an online support system for therapy assistance (CanKado) [38], and future trials will examine the impact of this system on breast cancer treatment. CanKado, which will be one of the first projects to provide additional patient support to breast cancer patients, is an electronic support system that aims to increase therapy success in oncology. Such technologies have the potential to increase compliance, improve doctor-patient relationships, and potentially even improve disease outcomes in the near future.

### Conclusion

This survey shows a high rate of Internet and modern media usage among physicians participating in breast cancer care. Online support, as well as online side effect registration, is favored by the majority of health care professionals surveyed. The routine usage of the Internet and modern media, and trust in new interactive communication tools, may enable improvements in doctor-patient relationships as well as in compliance and adherence among patients suffering from breast cancer.

Acceptance of such technologies by patients and other health care personnel involved in therapy management (eg, nurses) is also necessary. Moreover, the actual impact of new interactive media on oncological practice can only be investigated via trials that use newly-developed online platforms (eg, the CanKado-project [38]) for patient support. In conclusion, our results suggest that eHealth tools may have a promising future in patient-physician communication, and the treatment of breast cancer.

## Abbreviations

eHealth: electronic health
